# MicroRNA-503 acts as a tumor suppressor in glioblastoma for multiple antitumor effects by targeting IGF-1R

**DOI:** 10.3892/or.2013.2951

**Published:** 2013-12-30

**Authors:** YINGYING ZHANG, XIONG CHEN, HAIWEI LIAN, JIANMIAO LIU, BEIYAN ZHOU, SONG HAN, BIWEN PENG, JUN YIN, WANHONG LIU, XIAOHUA HE

**Affiliations:** 1 Hubei Province Key Laboratory of Allergy and Immunology, School of Basic Medical Sciences, Wuhan University, Wuhan, Hubei 430071, P.R. China; 2 Department of Neurosurgery, Renmin Hospital of Wuhan University, Wuhan, Hubei 430060, P.R. China; 3 College of Life Science and Technology, Huazhong University of Science and Technology, Wuhan, Hubei 430074, P.R. China; 4 Department of Physiology and Pharmacology, College of Veterinary Medicine and Biomedical Sciences, Texas A&M University, College Station, TX 77843, USA

**Keywords:** microRNA-503, insulin-like growth factor-1, glioblastoma, AKT, tumor suppressor

## Abstract

microRNA (miRNA) dysregulation is associated with various types of human cancer by regulating cancer cell survival, proliferation and invasion. Aberrant expression of microRNA-503 (miR-503) has been reported in several cancer profiles. However, potential linkage of miR-503 levels and the underlying regulatory mechanisms in human glioblastoma multiforme (GBM) remain unclear. In the present study, we showed for the first time that the expression of miR-503 was significantly reduced in GBM tissues and cell lines (U251 and U87MG) relative to normal brain tissues. Furthermore, our results demonstrated that overexpression of miR-503 in GBM cell lines not only suppressed cell proliferation through inducing G0/G1 cell cycle arrest and apoptosis, but also inhibited cancer cell migration and tumor invasion. In addition, we identified insulin-like growth factor-1 (*IGF-1R*) receptor mRNA is a bona fide target of miR-503 by computational analysis followed by luciferase reporter assays. Of note, upregulation of miR-503 in GBM cells suppressed endogenous IGF-1R protein expression. Further mechanistic analysis revealed that forced expression of miR-503 inhibited AKT activation, suggesting the tumor suppressive effect of miR-503 in GBM cells is partially mediated by phosphatidylinositol 3-kinase/AKT signaling. Taken together, the results of the present study demonstrated that miR-503 is a tumor suppressor for GBM and a favorable factor against glioma progression through targeting IGF-1R, thus providing a new evidence-supported prognostic marker for GBM diagnosis.

## Introduction

Glioma is the most common intracranial tumor accounting for ~60% of all intracranial tumors (1). According to World Health Organization (WHO) guidelines, glioma is divided into 4 grades. Among them, grade IV tumor refers to glioblastoma multiforme (GBM) that is the most aggressive form of glioma (2). Despite the urgent demand for treatment solutions against this malignant cancer, little is known about the causes or mechanisms of its cancerous transformation and progression.

There is ample evidence that microRNAs (miRNAs), a family of highly conserved small non-coding RNAs, have regulatory functions in cancer progression (3). In previous studies, our group demonstrated that miR-150 may present therapeutic strategies to treat MLL-AF9-related leukemia by regulating multiple oncogenes (4). Discovery of miRNAs provided a new path to understand the molecular mechanism of glioma (5). Ciafrè *et al* (6) and Chan *et al* (5) observed that miR-221 and miR-21 were significantly upregulated in GBMs using miRNA microarray analysis with patient samples, respectively. In contrast, miR-181a/b/c were downregulated in GBMs compared to the normal brain tissues. Notably, a group of miRNAs including miR-16 and miR-195, which belong to the miR-15/16 family involving miR-15a/b, miR-16, miR-195, miR-424 and miR-497, are downregulated in human glioblastoma cells, and their abnormal expression patterns are associated with the survival rate of GBM patients compared to non-tumorous cells (7–9). microRNA-503 (miR-503) is a member of the miR-15/16 family and it was first reported as a highly elevated miRNA in human retinoblastoma tissues using miRNA microarray analysis (10,11). However, the relative expression of miR-503 between GBM and normal brain as well as the function of miR-503 on GBM is unclear.

In the present study, we first analyzed the expression pattern of miR-503 in human GBM samples and cell lines followed by functional investigation of miR-503 in human GBM cell lines. Taken together, our results demonstrated that miR-503 is a tumor suppressor in GBM with multiple aspects of antitumor effects partially mediated by post-transcriptional downregulation of insulin-like growth factor-1 (IGF-1R) expression, thereby interfering with the PI3K/AKT pathway. These results elucidated a novel molecular mechanism for the pathogenic mechanism in glioma progression, and may thus provide novel support for the development of targeted therapy.

## Materials and methods

### Human tissue samples

All human normal brain and glioma tissues from patients were collected in the Department of Neurosurgery, Renmin Hospital of Wuhan University from 2011 to 2013. Normal brain tissues were obtained from patients with cerebral trauma. Glioblastoma tissues were obtained according to the diagnosis of clinical and pathological grading. Prior consent was obtained from all patients and the study was approved by the institutional research board.

### Cell culture and miRNA transfection

Human glioma cell lines U251 and U87MG were from ATCC (Manassas, VA, USA) and cultured according to methods previously described (7). Cells at 50–70% confluence were transfected with miR-503 mimics or non-specific mimics as negative control (NC) (RiboBio, Guangzhou, China) using Lipofectamine^®^ 2000 reagent (Invitrogen, Carlsbad, CA, USA), respectively.

### Bioinformatics and luciferase reporter assay

Target genes of miR-503 were first predicted using multiple target prediction algorithms: TargetScan (http://www.targetscan.org/) and miRanda (http://www.microrna.org/). The IGF-1R 3′ untranslated region (3′UTR) was amplified from human genomic DNA using PCR and cloned into pMIR-REPORT vector. The primers used were: forward, 5′-GGA CTA GTC TAG GAC TTC TTC ATG GGT CTT-3′ and reverse, 5′-ATG AAG CTT GTG TCA CAA CCT AAG CAA AG-3′. Twenty-four hours before transfection, U251 cells were seeded in a 24-well plate, the pMIR-REPORT vector bearing miR-503 binding site (IGF-1R-3′UTR-wt) or mutated binding site (IGF-1R-3′UTR-mut) constructs and pRL-TK vector were transfected using Lipofectamine 2000 reagent. Twenty-four hours after transfection, luciferase activities were evaluated using the Dual Luciferase Reporter Assay System (Promega, Madison, WI, USA) and the relative activity of *Renilla* luciferase were normalized to that of firefly luciferase harbored in the same reporter construct.

### RNA extraction and quantification assay

Total RNA from tissues and cell lines was extracted with TRIzol reagent (Invitrogen) and reverse-transcribed with RevertAid First Strand cDNA Synthesis kit (Fermentas, Vilnius, Lithuania). The primer sequences for IGF-1R gene expression were: IGF-1R forward, 5′-CCC AAG ACC CAG AAG GAA-3′ and reverse, 5′-ACT CGT GCA GAG CAA AGG AT-3′; GAPDH primer forward, 5′-CTT CAA CGA CCA CTT TGT-3′ and reverse, 5′-TGG TCC AGG GGT CTT ACT-3′. To analyze miR-503 expression levels, the Bulge-Loop™ miRNA qRT-PCR primer kits (RiboBio) were utilized according to the manufacturer’s instructions. RNA input was normalized to the level of human U6 snRNA. Real-time PCR was performed using All-in-One™ qPCR mix (GeneCopoeia, Guangzhou, China) with an iCycler thermal cycler (Bio-Rad). The 2^−ΔCt^ or 2^−ΔΔCt^ methods were used to calculate the relative expression level of miR-503 human tissues or the expression levels of miR-503 and IGF-1R in cell lines (n=3, means ± SEM).

### Proliferation assays

U251 and U87MG cells were seeded in 96-well plates with 6×10^3^ cells/well 24 h prior to the transfection of miR-503 mimics or NC and assayed 24, 48 and 72 h after transfection. Twenty microliters of dimethyl thiazolyl diphenyl tetrazolium bromide (MTT) (5 mg/ml) were added into each well and the incubation continued for 4 h. Then, supernatant was removed and 100 μl dimethyl sulfoxide (DMSO) was added to each well to dissolve the precipitate. Optical density (OD) value was measured at the wavelength of 570 nm.

To further evaluate cell proliferation, cells transfected with miR-503 mimics or NC were cultured for 20 h, and then incubated for 4 h with DMEM containing 1 mCi/ml [^3^H]thymidine (Amersham, Shanghai, China). The cells were washed with ice-cold PBS, fixed with ice-cold 10% trichloracetic acid, and solubilized by 0.3 M NaOH. The incorporated radioactivity was quantified using a Beckman scintillation counter.

### Apoptosis assay

U251 and U87MG cells were transfected with miR-503 mimics or NC for 48 h. Cells were harvested in cold phosphate-buffered saline (PBS), stained with Annexin V-FITC and propidium iodide (PI) for 10 min and analyzed by flow cytometry (EPICS Altra II; Beckman) and date SPSS version 17.0.

### Cell cycle assay

Forty-eight hours after transfection, U251 and U87MG cells were harvested in ice-cold PBS and then fixed with 70% ice-cold ethanol for an additional 48 h at 4°C. The fixed cells were incubated with PI for 30 min at 37°C in the dark. The DNA content was analyzed by flow cytometry.

### Wound healing assay

Transfected U251 cells were seeded in 6-well plates at a density of 2×10^5^ cells/well. At 80% confluence the cells were scratched with a 10 μl plastic pipette tip to form a straight wound and cultured for an additional 48 h. The wound closure was measured under a microscope equipped with a camera. Images of 3 random fields were captured at the time of 0, 24 and 48 h after wounding.

### Cell migration and invasion assays

The details of cell migration and invasion assay methods used are as previously described (7). Twenty-four-well Transwell plates (8-μm pore size) (Corning, New York, USA) were used for these experiments. Cells (3×10^4^) were plated in the top chamber for migration or invasion assay. The migration and invasion rates were measured by photographing at 5 random fields.

### Protein extraction and western blotting

Forty-eight hours after transfection, cellular proteins were extracted with lysis buffer supplemented with protease inhibitors. The supernatant of the lysate was collected and separated by SDS-PAGE, then transferred to PVDF membranes. The proteins were respectively probed with primary antibodies, incubated with HRP-conjugated secondary antibodies and then visualized with an ECL detection system. Protein expression was measured by ImageJ software.

### Statistical analysis

Data are presented as mean values ± SEM, and were analyzed by use of SPSS version 17.0 (SPSS, Chicago, IL, USA). Significance between two group comparisons were analyzed with a t-test, and relationships between more than two group comparisons were analyzed with one-way ANOVA. p<0.05 was considered to indicate a statistically significant difference. All experiments were carried out in triplicate unless otherwise noted.

## Results

### miR-503 is downregulated in human GBM tissue samples and cell lines compared to normal brain tissues

**Figure 1:**
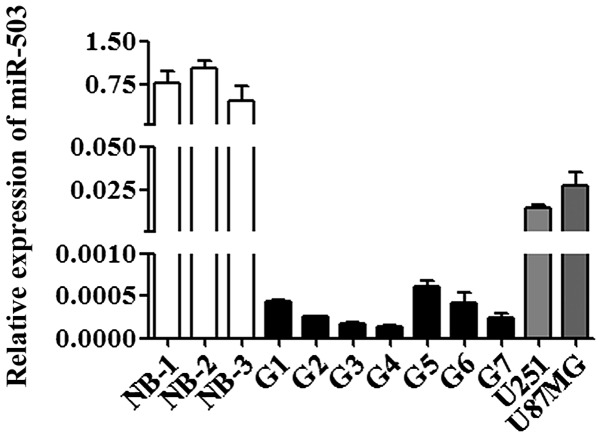
miR-503 expression is suppressed in glioma tissues and cell lines compared to normal brain tissues. Real-time PCR analyzed miR-503 expression in 3 normal brain tissues (NB1, NB2 and NB3), 7 glioma tissues (G1–G7) and 2 glioma cell lines (U251 and U87MG). RNA input was normalized by human U6 snRNA. The 2^−ΔCt^ method was used to calculate the miR-503 expression. miR-503, microRNA-503.

To evaluate the expression pattern of miR-503, we performed quantitative RT-PCR analysis. The level of miR-503 was significantly lower in human GBM tissue samples compared to normal brain tissues (p<0.001; Fig. 1). This is similar to the suppressed expression level of miR-503 in human GBM cell lines (U251 and U87MG) (p<0.001; Fig. 1), suggesting a potential tumor-suppressive function of miR-503 in GBM.

### Overexpression of miR-503 inhibits the propagation of GBM cell lines in vitro

**Figure 2:**
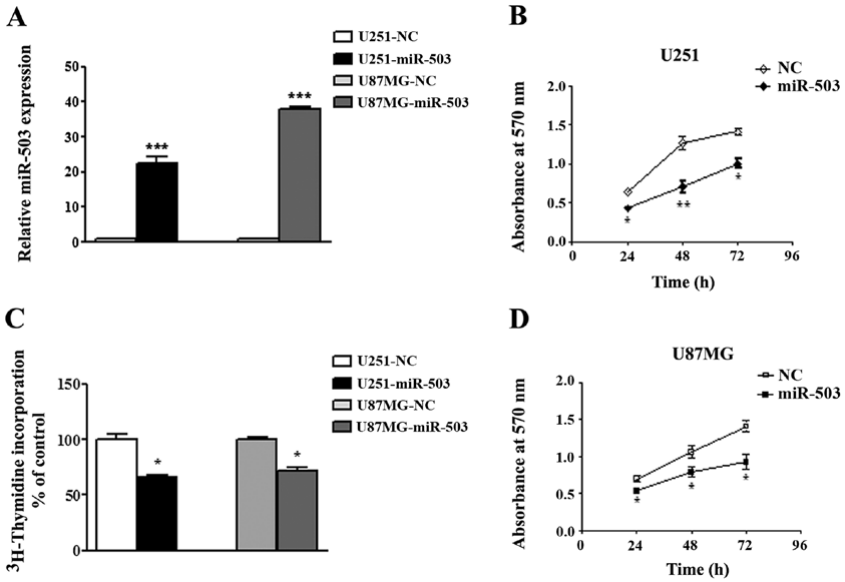
Overexpression of miR-503 inhibits cellular proliferation in U251 and U87MG cells. (A) The expression of mature miR-503 was detected in both GBM cell lines after transfection with miR-503 mimics or negative control for 48 h. ^***^p<0.001. (B and D) Overexpression of miR-503 inhibited the propagation of U251 and U87MG cells by MTT assay. ^*^p<0.05, ^**^p<0.01. (C) Overexpression of miR-503 increased thymidine uptake in U251 and U87MG cells. ^*^p<0.05. miR-503, microRNA-503; GBM, glioblastoma multiforme.

To understand the function of miR-503 in GBM, we utilized both gain of loss of function strategies in the cultured cells. U251 and U87MG cells were transiently transfected with miR-503 mimics or NC and efficiency of the transfection was examined by real-time PCR assays at 48 h after transfection (Fig. 2A). Next, the effect of forced expression of miR-503 on cell proliferation was evaluated using MTT assays at indicated time points. As expected, transfection of both cell lines with miR-503 mimics oligonucleotides resulted in a marked suppression of cell growth activity compared to cells transfected with NC oligos after 24 h (p<0.05; Fig. 2B and D), 48 h (p<0.01 in U251 and p<0.05 in U87MG; Fig. 2B and D) and 72 h (p<0.05; Fig. 2B and D). This inhibitory effect of ectopic expression of miR-503 was further confirmed using a [^3^H]thymidine incorporation assay (p<0.05; Fig. 2C). Taken together, these results demonstrated an antiproliferative effect of miR-503 on GBM cell lines.

### miR-503 induces G0/G1 phase arrest in cell cycle distribution

**Figure 3:**
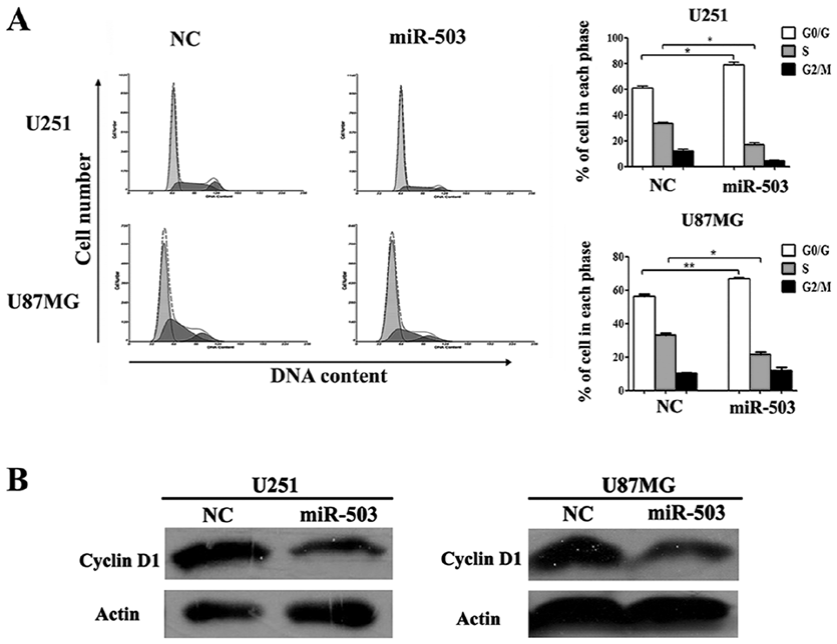
miR-503 blocks cell cycle progression in the S phase of U251 and U87MG cells. (A) U251 and U87MG cells were transfected with miR-503 and NC for 48 h, cell cycle distribution was detected by FACS. Histograms showed G0/G1 phase arrest and S phase decline in both cell lines transfected with miR-503 mimics. ^*^p<0.05; ^**^p<0.01. (B) Analyzed by western blotting, the level of CCND1 protein expression was decreased. miR-503, microRNA-503; NC, negative control; CCND1, cyclin D1.

To further define the antiproliferative ability of miR-503 in glioblastoma cells, we analyzed cell cycle distribution in GBM cells transfected with miR-503 mimics or NC control oligos using flow cytometry analysis. As shown in Fig. 3A, the G0/G1 phase fraction of cells with NC control was 60.97±1.16% (n=3, in U251 cells) and 56.43±1.04% (n=3, in U87MG cells), whereas cells transfected with miR-503 mimics increased the percentage of cells in G0/G1 phase 79.07±1.91% (n=3, in U251 cells) and 66.77±0.88% (n=3, in U251 cells). Moreover, the average S phase fraction in cells with miR-503 appeared significantly lower (mean, 16.40±1.82%, n=3, in U251 cells and mean, 11.73±2.67%, n=3, in U87MG cells) compared to NC-treated cells. Furthermore, the protein level of cyclin D1 (CCND1) which served as an active switch in the regulation of cell cycle during G1/S transition displayed a significant reduction observed by western blot analysis in both cell lines (Fig. 3B). These results suggested that overexpression of miR-503 induced G0/G1 arrest, thereby delaying the progression of cell cycle.

### miR-503 enhances apoptosis of glioblastoma

**Figure 4:**
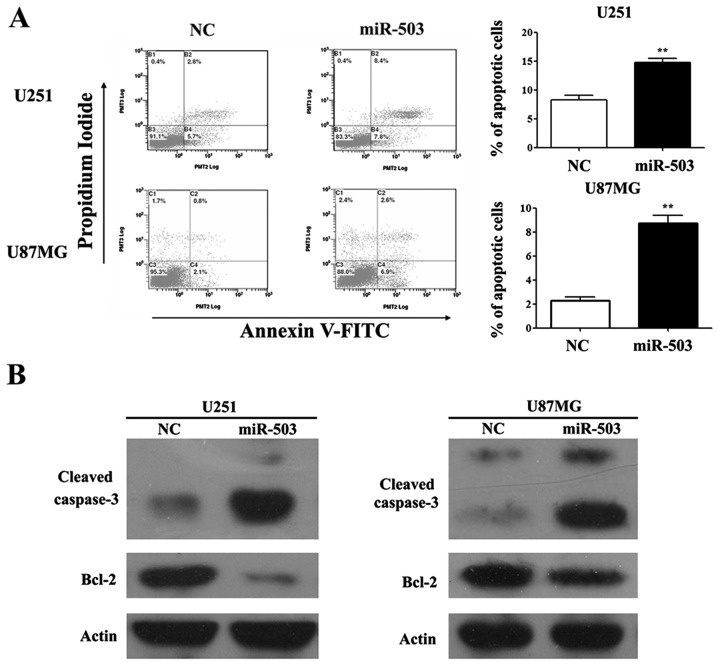
miR-503 induced apoptosis of U251 and U87MG cells. (A) Apoptosis was measured by Annexin V-FITC/propidium iodide (PI) staining following miR-503 mimics or normal control treatment. Histograms showed that total apoptosis rate of 2 glioblastoma cell lines, containing early and late apoptosis rates, was significantly increased after transfection with miR-503 mimics. ^**^p<0.01. (B) Increased cleaved caspase-3 protein expression and decreased Bcl-2 protein expression were observed in 2 cell lines by western blot assay. miR-503, microRNA-503.

To evaluate the effects of miR-503 on GBM cancer cell survival, we adopted cell apoptosis assays in both U251 and U87MG cell lines. Forty-eight hours after transfection of miR-503 mimics or NC, cells were collected and analyzed for the binding of Annexin V and PI penetration using flow cytometry. A markedly induced cell apoptotic rate defined as the proportion of Annexin V^+^PI^+^ population was observed in cells transfected with miR-503 mimics compared to that of cells with NC oligo transfection (p<0.01; Fig. 4A). Moreover, we also detected an increased level of cleaved caspase-3 protein and Bcl-2 protein in cells with elevated miR-503 levels compared to NC (Fig. 4B).

### miR-503 inhibits cell migration and invasion of glioblastoma cells

**Figure 5:**
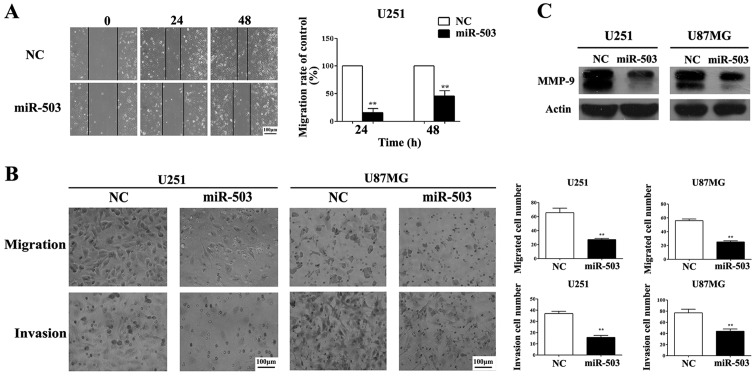
miR-503 inhibits migration and invasion of U251 and U87MG cells. (A) The wound healing assay showed delayed closure in miR-503-transfected cells compared with normal control at the 24 and 48 h time points in U251 cells. The mean percentage of wound closure at 24 and 48 h after wounding were significantly decreased in U251 cells transduced with miR-503. ^**^p<0.01. (B) Representative images subjected to the migration and invasion assay. Overexpression of miR-503 significantly impeded cell migratory ability and invasion ability of U251 and U87MG cells. ^**^p<0.01. (C) Western blot assay showed that MMP-9 protein expression was inhibited in 2 cell lines transfected with miR-503 mimics. miR-503, microRNA-503.

Based on our results of MTT and flow cytometry (Figs. 2 and 4), we hypothesized that loss of miR-503 in GBM cells may contribute to the invasion of GBM tumor. To test this hypothesis, we performed a set of experiments to evaluate cell migration and tumor invasion. First, we adopted cell migration analysis using Transwell methods. U251 and U87MG cells transfected with miR-503 mimics displayed a substantially suppressed migratory and invasive capacity compared to their respective control groups (Fig. 5B). As expected, the inhibitory effect of miR-503 on cancer cell invasion was further confirmed using the wound healing assays (Fig. 5A). Furthermore, we observed a decrease in the MMP-9 protein level, a cell migration regulator in gliomagenesis (12), in cells with elevated miR-503 compared to control samples as shown by western blot assays, suggesting a potential mechanism of miR-503 inhibiting tumor invasion (Fig. 5C).

### IGF-1R is a direct functional target of miR-503 that partially mediates the effect of miR-503 through AKT activation in glioblastoma cells

**Figure 6:**
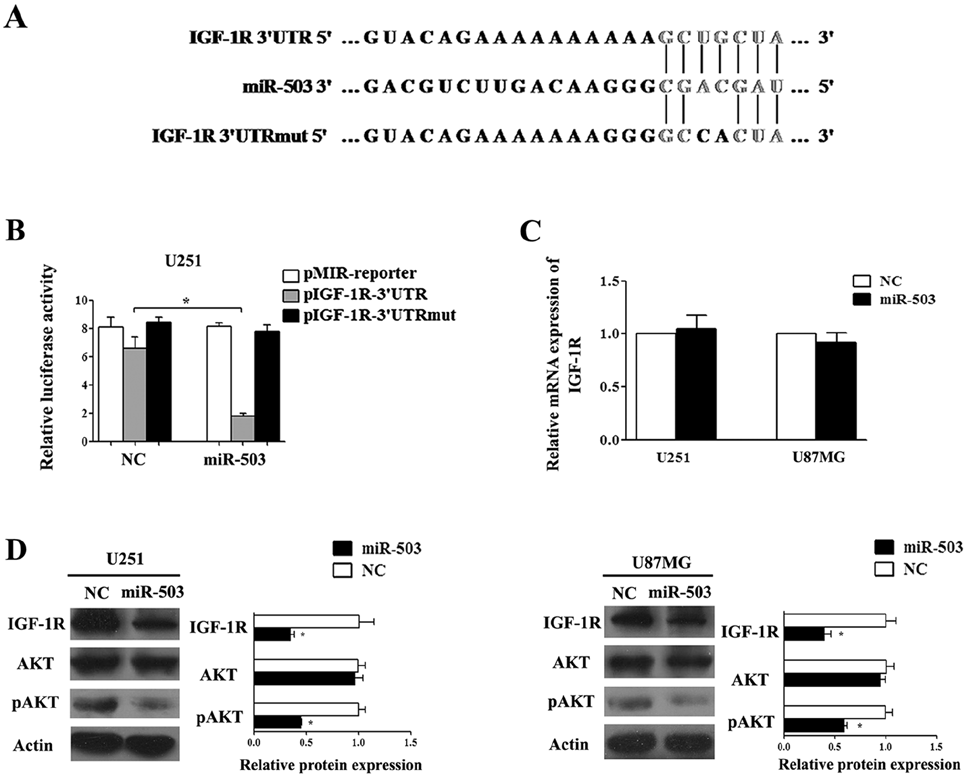
miR-503 directly targets IGF-1R and downregulates IGF-1R protein expression in U251 and U87MG cells. (A) Wild-type and mutant of putative miR-503 target sequences of IGF-1R 3′UTR. ^*^p<0.05. (B) Luciferase reporter assays showed that miR-503 suppressed the luciferase activity of wild-type IGF-1R 3′UTR. (C) Analyzed by real-time PCR, miR-503 had no effect on the mRNA expression of IGF-1R. (D) Western blot assay showed that miR-503 inhibited IGF-1R protein expression and phosphorylation of AKT at Ser473. Data are means of 3 independent experiments. ^*^p<0.05. miR-503, microRNA-503; IGF-1R, insulin-like growth factor-1; 3′UTR, 3′ untranslated region.

To investigate the responding molecular components in miR-503 tumor-suppressive regulation, we sought to identify the direct target gene in the context of glioblastoma. Surveying the online algorithms miRanda and TargetScan, we found that IGF-1R, a central receptor protein to facilitate tumor development mainly by AKT and MAPK pathways, bears miR-503 target site in its 3′UTR region, thus may serve as a potential target gene of miR-503 (Fig. 6A). To determine direct regulatory binding of miR-503 on IGF-1R, we constructed a luciferase reporter assay using pMIR-REPORT constructs containing a segment of the 3′UTR with wild-type or mutated seed region. Luciferase assays demonstrated that miR-503 suppressed activity of luciferase in constructs carrying the wild-type target sites but not the site with point mutations (Fig. 6B). Next, we examined the mRNA and protein levels of IGF-1R in U251 and U87MG cells using real-time PCR and western blot analysis. Our results showed that the level of IGF-1R protein was markedly decreased but there was no apparent change in the IGF-1R mRNA levels when miR-503 was overexpressed (Fig. 6C and D), suggesting miR-503 was involved in the regulation of IGF-1R expression.

The activation of the AKT pathway is crucial for the sensitivity of glioblastoma cells to IGF-1R inhibitors (13). Therefore, we examined both levels of total AKT (AKT) and phosphorylated AKT (pAKT) proteins in U251 and U87MG cells. Western blot results demonstrated that treatment with miR-503 mimics reduced the expression of phosphorylated AKT, whereas the effect of miR-503 on total AKT protein level was not statistically significant (Fig. 6D). Collectively, these results suggest that miR-503 may suppress GBM progression probably by inhibiting the IGF-1R/PI3K/AKT pathway.

## Discussion

In the present study, we found that miR-503 was downregulated in GBM tissues and cell lines related to normal brain tissues. Moreover, introduction of exogenous miR-503 inhibited cell growth, migration and invasive ability, induced G0/G1 phase arrest and enhanced apoptosis of U251 and U87MG human glioblastoma cells. Further, we identified IGF-1R as a direct functional target of miR-503 which exerts important effects on glioblastoma cells. These results support an antineoplastic role for miR-503 in GBMs.

It is of note that the expression patterns of miR-503 vary in different tumor tissues and cell lines and its specific impact on the tumor biology needs to be further investigated. miR-503 was overexpressed in human retinoblastoma tissues as detected by miRNA microarray analysis. Corbetta *et al* (11) also observed the phenomenon in human parathyroid carcinomas and adenocortical carcinomas (15,16). On the contrary, Zhou *et al* reported that miR-503 was markedly downregulated in primary HCC tissues compared to their adjacent non-cancerous liver tissues (17) and a similar observation was reported in endometrioid endometrial cancer and cisplatin-resistant non-small cell lung cancer cells (18–20). In our study, we found that miR-503 was significantly downregulated in GBM tissues and two glioma cell lines compared to three normal brain tissues. A putative reason for such tumor-specific expression patterns of miR-503 may be due to the feedback adjustment, tumor progression stages and diversity of internal environment between different types of tumor.

miR-503 is a member of the miR-15/16 family and all the family members shared a common characteristic of 5′-end AGCAGC motifs in the mature miRNA (21). As some group members of the miR-15/16 family have similar sequences and consistent expression profiles in different human tissues, they may have similar or synergistic effects in a certain pathogeneses (22–24). For example, downregulation of miR-195 and miR-497 may strongly affect cell cycle progression and lead to an aberrant cell proliferation in hepatocellular carcinoma cell lines (25). Given that miR-16-1 and miR-195 played tumor-suppressor roles in human glioblastoma cells, we hypothesized that miR-503 may have a similar antitumor effect as other members of the miR-15/16 family in human glioblastoma cells (7,8). Consistent with this hypothesis, we found that elevated miR-503 level not only suppressed cellular proliferation, inhibited cell migration and invasion, but also enhanced apoptosis in U251 and U87MG cells.

miR-503 may also be a potent cell cycle regulator by targeting multiple cell cycle-related proteins. Caporali *et al* (26) and Sarkar *et al* (27) identified CCNE1 and CDC25A as direct targets of miR-503 in endothelial cells and osteosarcoma cells respectively, leading to the block in G0/G1 and G2/M phase transitions. Moreover, Jiang *et al* demonstrated that miR-503 may reduce S phase cell populations by targeting CCND1 3′UTR and resulted in human head and neck carcinoma cell growth inhibition (28). Our results further confirmed that miR-503 may suppress the endogenous CCND1 protein level and induce G0/G1 phase arrest in GBM cell lines. These results suggested that miR-503 may play a key role in cell cycle regulation. The progression of glioma is correlated with increased expression level of IGF-1R (29). IGF-1R is known to play a pivotal role in regulating cell proliferation and survival in the process of gliomagenesis (30–32). IGF-1R exerts its effect primarily through activating MAPK kinases and the PI3K/AKT pathways, that may further influence the invasion of glioma cells (33,34). Moreover, recent studies demonstrated that a number of cell cycle-related and invasion-associated genes are regulated by IGF-1R, including CCND1 and MMP-2/MMP-9 in human glioma and head and neck cancer, which function through MAPK and PI3K/AKT pathways (35–41). In the present study, we found that IGF-1R was a direct target of miR-503 and indicated the IGF-1R/PI3K/AKT pathway may contribute to the biological effects of miR-503.

**Figure 7:**
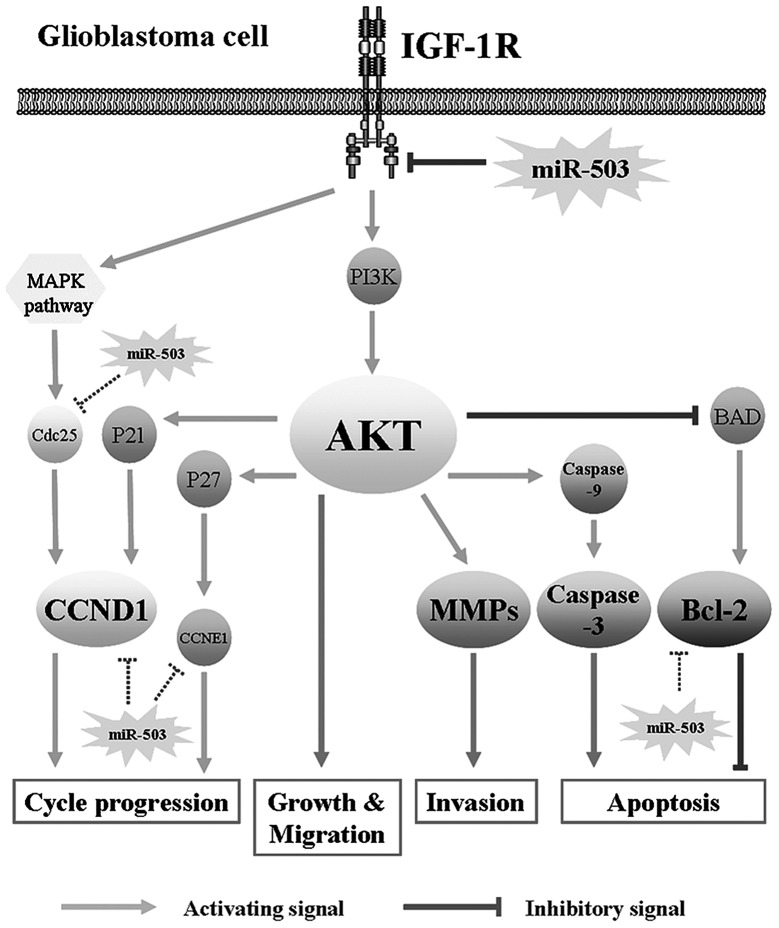
Model of miR-503 mediates constitutive suppression of the IGF-1R pathway leading to a less aggressive glioma phenotype. The IGF-1R/AKT pathway is a core signaling pathway in glioblastoma oncogenesis. miR-503 influenced AKT pathway by targeting IGF-1R, and consequently inhibits cell growth, cell cycle progression, cell migration and invasion, and induces cell apoptosis. Among them, AKT may regulate cell cycle progression by P21/CCND1, influence cellular invasiveness by MMP-9, affect apoptosis by caspase-3 and Bcl-2. In addition to directly target IGF-1R, miR-503 may also influence GBM pathogenesis by directly regulating Bcl-2, CDC25, CCND1 and CCNE1 according to previous research. miR-503, microRNA-503; IGF-1R, insulin-like growth factor-1; GBM, glioblastoma multiforme.

To the best of our knowledge, this is the first description of the tumor-suppressive role of miR-503 in GBMs and its functions through regulating IGF-1R and its AKT activation, leading to potent suppression in cancer cell proliferation, survival, migration and invasion. In summary, we conceived a specific mechanism of miR-503 regulatory impacts on a variety of tumors, including the current discovery in glioblastoma (Fig. 7). In combination with previous and current studies, we highlighted a potential regulatory mechanism of miR-503 on cell cycle and apoptosis-related protein in GBM cells (26,28,42). Collectively, our study provided a possible therapeutic application against glioma by a gene therapy approach with introduction of exogenous miR-503.
